# Predicting Landscape-Genetic Consequences of Habitat Loss, Fragmentation and Mobility for Multiple Species of Woodland Birds

**DOI:** 10.1371/journal.pone.0030888

**Published:** 2012-02-17

**Authors:** J. Nevil Amos, Andrew F. Bennett, Ralph Mac Nally, Graeme Newell, Alexandra Pavlova, James Q. Radford, James R. Thomson, Matt White, Paul Sunnucks

**Affiliations:** 1 School of Biological Sciences and Australian Centre for Biodiversity, Monash University, Melbourne, Victoria, Australia; 2 Landscape Ecology Research Group, School of Life and Environmental Sciences, Deakin University, Burwood, Victoria, Australia; 3 Arthur Rylah Institute for Environmental Research, Department of Sustainability and Environment, Heidelberg, Victoria, Australia; Biodiversity Insitute of Ontario - University of Guelph, Canada

## Abstract

Inference concerning the impact of habitat fragmentation on dispersal and gene flow is a key theme in landscape genetics. Recently, the ability of established approaches to identify reliably the differential effects of landscape structure (e.g. land-cover composition, remnant vegetation configuration and extent) on the mobility of organisms has been questioned. More explicit methods of predicting and testing for such effects must move beyond *post hoc* explanations for single landscapes and species. Here, we document a process for making *a priori* predictions, using existing spatial and ecological data and expert opinion, of the effects of landscape structure on genetic structure of multiple species across replicated landscape blocks. We compare the results of two common methods for estimating the influence of landscape structure on effective distance: least-cost path analysis and isolation-by-resistance. We present a series of alternative models of genetic connectivity in the study area, represented by different landscape resistance surfaces for calculating effective distance, and identify appropriate null models. The process is applied to ten species of sympatric woodland-dependant birds. For each species, we rank *a priori* the expectation of fit of genetic response to the models according to the expected response of birds to loss of structural connectivity and landscape-scale tree-cover. These rankings (our hypotheses) are presented for testing with empirical genetic data in a subsequent contribution. We propose that this replicated landscape, multi-species approach offers a robust method for identifying the likely effects of landscape fragmentation on dispersal.

## Introduction

Habitat loss and fragmentation lead to small and increasingly isolated populations of wildlife in habitat remnants, decreasing metapopulation viability [1]–[3]. Small, isolated populations lose fitness through inbreeding depression of individuals and loss of genetic diversity from populations, decreasing adaptability to environmental change; these processes elevate extinction risk [4]–[6]. If the mean probability of extirpation in remnants exceeds the mean probability of recolonisation, then metapopulation extinction will eventuate. The time lag over which this occurs depends on many factors and may be many generations [7]. This ‘extinction debt’ is the number of taxa that, following habitat loss, no longer satisfy a threshold criterion for their survival [8]. Thus many authorities [9], [10] have identified the critical role of connectivity (the inverse of fragmentation) at landscape, regional and continental scales in effective conservation management.

An ongoing challenge is to tease apart the often interrelated ecological and genetic processes that result in biodiversity loss following habitat loss and alteration [11], [12]. Such knowledge is essential in order to design and implement management interventions to ‘repay’ extinction debt before species are lost [13].

### Landscape-genetic approaches to assessing effects of habitat alteration

Landscape genetics [14] when combined with spatial modelling [15] provides techniques for linking observed patterns of species' occurrence to processes, particularly the relationships among structural and functional connectivity [16], [17], genetically effective dispersal [18] and the maintenance of populations in fragments. Typically, landscape-genetic studies have involved *post hoc* fitting of models to explain the relationship between genetic patterns and landscape structure. But this approach is limited in the robustness of its predictions, because alternative connectivity models are frequently correlated [19]. Further, such models have usually been limited to inferences about a single species [20].

*A priori* statements of explanatory models offer a more rigorous approach to linking observed pattern with process [19], [21], [22]. Replicate testing of predictions across multiple landscapes and species greatly strengthens inferences about population processes by testing generality [23]. The need for replication in landscape-genetic studies has been emphasised in recent reviews [19], [24], [25]. Inferences can be reinforced by concurrent examination of sympatric species predicted to have different responses to fragmentation on the basis of their known ecology and behaviour [26]. This approach is valid even where relatively little is known about species' attributes [27], [28].

### Modelling ‘Effective Distance’ for comparison with genetic data

Structural connectivity is an attribute of the physical configuration of suitable habitat patches within a landscape. Functional connectivity is an emergent property of individual species-landscape interactions [17]. It has been defined as ‘the degree to which the landscape facilitates or impedes movement among resource patches’ [16], and thus reflects the effect that landscape structure and different landscape elements have on the dispersal ability and gene flow of an organism [29]–[31].

The most widely adopted approach to estimating the relationship between structural and functional connectivity is to model ‘effective distance’, the “Euclidean distance modified for the effect of landscape and behaviour” [32] on the dispersal of an organism between locations in the landscape. Effective distance can then be compared with dissimilarity or distance measures, such as genetic distances between populations or individuals, or estimates of numbers of dispersers between habitat patches in a landscape.

Effective distance may be modelled by using least-cost path algorithms [32], [33]. These account for differing costs (resistance per unit distance) of passing through different landscape elements. The algorithms identify the path through a landscape that minimizes the resistance to an organism moving between two points, and thus calculate the least-cost distance. Such information on potential paths through the landscape, correlated with estimates of functional distances or dispersal (e.g. genetic distances or observed dispersal events from mark-release-recapture or radiotelemetry), is often used to estimate the role of landscape structure as a constraint to dispersal [34]–[37].

Least-cost path modelling has been criticized for its biologically unrealistic assumptions, such as that the disperser has complete prior knowledge of its surroundings and on this basis chooses the least costly path [29], [38]. Another perceived drawback is that simple least-cost path analysis identifies only a single optimal route, rather than the contribution of multiple possible routes to effective distance [38], and so may not represent gene flow which accumulates across multiple dispersal events over time. Despite its limitations, least-cost path modelling has consistently shown predictive value when tested with molecular-genetic data [30], [37], [39], [40] and compared with dispersal paths derived from radiotelemetry [41].

Extensions of least-cost path methods may partially overcome some of these limitations by allowing the mapping of near-optimal or multiple pathways [38], [42], [43]. The isolation-by-resistance model of McRae [44], also based on calculations of movement costs across a resistance surface, is becoming more widely adopted [45]. Isolation-by-resistance offers a conceptual model in which landscape resistance is the analogue of electrical resistance, and the movements of individuals and flow of genes are analogues of electrical current. It greatly extends the ability to model multiple complementary paths of connectivity, while being sufficiently computationally efficient to allow its use over large landscapes at relatively fine resolution (e.g. grids of 10^8^ cells) [46]. The associated software, Circuitscape [47], generates maps of current (an analogue of gene flow or dispersal density) that indicate potentially important areas for maintenance of, or constraints to, functional connectivity.

Isolation-by-resistance was found to explain a greater proportion of variance in genetic population structure than isolation-by-distance or least-cost distance in simple model networks and when dealing with species' ranges at (sub)continental scales [40], [44]. At least one other study found that least-cost distance explained a greater proportion of genetic variation than circuitscape distance; however, the resolution of the grids used in the two calculations were different [48]. The present study builds on this single comparison of the two approaches by examining their performance across multiple species in the same landscapes.

Here, we construct a set of landscape resistance surfaces for use in modelling effective distance, to represent a number of alternative hypotheses about gene-flow. This work forms part of a related large-scale empirical study in which we collected genetic data from 10 species of woodland bird, sampled at 65 sites across 12 landscapes (each 100 km^2^) that differ in their extent and configuration of wooded native vegetation. In a subsequent contribution, we test these predictions generated from these gene flow hypotheses using empirical genetic data at two spatial scales: (1) relatively short distances *within* replicated landscapes; and (2) greater distances *across* the whole study area.

We take the approach advocated by Cushman and Landguth [49] of incorporating multiple alternative hypotheses of genetic differentiation, ranging from no spatial structuring, through isolation-by-distance [50], to a number of alternatives representing heterogeneous landscape resistance. Based on these alternative hypotheses (represented by different resistance surfaces) we calculated effective distances between all sample collection sites, using two of the main methods for estimating effective distance: least-cost path analysis, and isolation-by-resistance using Circuitscape [46]. We also identify the appropriate null model representing isolation-by-distance in a uniform landscape for each [51]. Correlations between each effective distance model and the relevant null model are reported to emphasize potential challenges in distinguishing these effective distance models from pure isolation-by-distance [51]. For each target species, we rank *a priori* the expectation of fit of genetic response to the effective distance models according to the expected response of birds to loss of structural connectivity and landscape-scale tree-cover. These expectations will later be tested using partial Mantel tests and ‘causal modelling’ [52], [53]. Causal modelling is a technique to alternately condition each of two dissimilarity matrices using the other to examine the residual effect of each matrix on a third matrix in a series of Mantel and partial Mantel tests [53].

Very different inferences about landscape resistance may result from resistance model tests in fragmented and unfragmented landscapes [54]. Our study design contains landscapes at three levels of fragmentation and varying levels of cover in fragmented landscapes for further exploration of this problem.

Several studies of landscape connectivity with both genetic data and individual tracking have used model selection between multiple landscape resistance hypotheses [28], [53]–[56]. Some have strengthened their inferences by replication of landscapes, and one considered two species with contrasting habitat and *a priori* expectations of response to fragmentation [28]. The multiple model selection approach reduces the probability of affirming the consequent [49] where the range of plausible resistance hypotheses are incorporated in the models chosen. Landscape replication further reduces the chances of misleading correlations resulting from configuration of samples and landscape elements in a single landscape [25].

### Woodland birds of the Box-Ironbark forests of central Victoria, Australia

The avifauna of dry woodland systems of southern Australia is experiencing continuing decline, due primarily to habitat loss compounded by a range of other contributory factors [57]–[59]. Radford et al. [60] examined the incidence of 58 species of woodland-dependent bird in remnant tree-cover in 24 landscapes, each 10×10 km^2^ in central Victoria. Below a threshold of c. 10% of native tree-cover, there were steep declines in landscape-level species richness. Radford et al. [60] interpreted this threshold in species richness as the terminal point of a series of species-level declines that commenced at much higher levels, c. 30–50%, of vegetation cover, indicating evidence of local payment of the extinction debt. There was much variation in the landscape attributes identified as most influential in predicting the incidence of individual species at the landscape scale and in the shape of individual species' responses to landscape level tree-cover. About one-third of species showed no significant relationship between incidence in the landscape and level of tree-cover, while other species showed a curvilinear response, indicating that these species' occurrences were declining more rapidly than expected given relative tree-cover [61], [62].

We examined current understanding of the mobility of 10 bird species to construct predictions of the effects of habitat loss and fragmentation. We constructed hypotheses about the extent to which the level of structural connectivity is reflected in changes in functional connectivity that might be signalled by changed gene flow. In a subsequent paper we will test the predictions generated from these gene flow hypotheses using empirical genetic data, and examine some of the possible causes that may explain the pre-identified patterns of decline.

In summary, our intentions in this paper are to:

1. Assemble and apply biological data and expert opinion to characterize the expected mobility of a suite of birds through different land-cover classes in our study system.
2. Formulate species-specific and spatially-explicit prior models of gene flow (represented by pairwise effective distances), and rank them for each species, to yield explicit prior hypotheses of gene flow for subsequent testing with genetic data.
3. Use and compare two predominant approaches to modelling effective distance (and hence connectivity), least-cost path analysis and isolation-by-resistance, including validation of the most appropriate null models for each.

## Materials and Methods

### Ethics statement

Observation of birds was carried out under DSE/DNRE permit numbers 10004294 and 10002099 under the Wildlife Act 1975 and the National Parks Act 1975, DSE permit number NWF10455 under section 52 of the Forests Act 1958 with approval and monitoring of Monash University ethics processes (BSCI/2007/07).

### Study area

**Figure 1:**
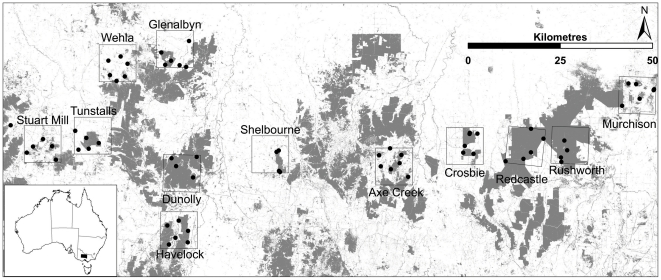
The study area in central Victoria, Australia, showing landscapes, sampling sites and remnant tree cover (shaded). Values for landscape treecover (%) are: 1. Landscapes with aggregated tree cover; Shelbourne 12%, Glenalbyn 17%,Tunstalls 20%, Crosbie 26% Havelock 45%. 2. Landscapes with dispersed tree cover; Welha 11%, Stuart Mill 19%, Murchison 27%, Axe Creek 35%, 3. Landscapes with continuous tree cover; Redcastle 75%, Dunolly 79%, Rushworth 79%.

The study area is c. 10,000 km^2^ of central Victoria in south-eastern Australia (Figure 1). The remnant native vegetation of the area is principally Box-Ironbark forest dominated by Grey Box (*Eucalyptus microcarpa*), Red Ironbark (*E. tricarpa*) and Yellow Gum (*E. leucoxylon*) on relatively infertile soils. Grassy forest and woodland containing *E. microcarpa*, *E. leucoxylon* and Yellow Box (*E. melliodora* ) remnants occur on more fertile valley floors, with River Red Gum (*E. camaldulensis*) dominant along watercourses. These latter vegetation types were selected for pastoralism in the 1840s, and much of the landscape has been cleared of native woody vegetation for >100 years. During the gold rushes of the 1850s–1860s, considerable logging and clearing of the native forests occurred and <2% of remaining forests are old growth [63]. Land-clearing for agriculture followed, alongside timber-cutting and firewood harvesting from 1870 to the Second World War and beyond [63]. Consequently, remnant forests and woodlands of the region are heavily fragmented, degraded and of low productivity. Only 19.2% tree-cover remains in the study area [64]. The intervening land is heavily cleared, though scattered trees remain in parts of the farmland [63].

### Landscape and site selection

Twelve 10×10 km landscapes were selected, nine among those used by Radford and Bennett [60], [61]. The present study aimed to identify processes leading to species declines. Therefore, all selected landscapes had tree-cover above the 10% threshold proposed by Radford et al. [60]. The landscapes represented two tree-cover configuration classes, ‘dispersed’ or ‘aggregated’ [60]. Three other ‘reference’ landscapes were selected with the highest available extant tree-cover (72–78%) to approximate continuous tree-cover (Figure 1). Reference landscapes necessarily contain a high proportion of Red Ironbark forest, because of the selective clearance of vegetation types across the region [63]. Sample sites within these landscapes were chosen to be as similar as possible in local vegetation type to the fragmented landscapes.

All landscapes were composed of six land-cover classes in varying proportions. The classes were: native tree-cover, plantation and horticulture, urban, unimproved pasture and native grassland, improved pasture and arable land. The last three land-cover classes further subdivided according to presence or absences of scattered trees.

Within each landscape, 3–6 sites were selected for genetic sampling. Initial sites were chosen at the locations of transects used by Radford et al. [60] in which there had been multiple incidences of the majority of the 10 target species (see below). The remaining sites were chosen to make possible the capture of a reasonable sample of the target species, and to provide a range of between-site distances.

### Study species

Our study design compared ‘decliner’ species (i.e., ones in which landscape-level incidence decreased disproportionately relative to landscape-level tree-cover) with ‘tolerant’ species (i.e., landscape-level incidence was proportionate to landscape-level tree cover). We analysed responses of 58 woodland-dependant species to landscape tree-cover from data in Radford [61] to classify them as decliner or tolerant to decreasing area of treecover (Material S1, Table S1).

We then applied two filters to select a subset of these 58 species as study species. First, species had to be common enough in the study landscapes that there was a high likelihood of obtaining sufficient samples for genetic analysis from multiple sites. Second, we stratified species by assumed mobility from highly mobile to sedentary. Data to classify relative mobility were collated from the standard reference work on the avifauna of Australia [65], [66]. These data collectively were used to categorize mobility subjectively for each species as sedentary, intermediate or mobile.

Ten study species were chosen (Table 1): two ‘tolerant’ species; White-plumed Honeyeater (*Lichenostomus penicillatus*), Striated Pardalote (*Pardalotus punctatus*), and eight ‘decliners’ – Brown Treecreeper (*Climacteris picumnus*), Eastern Yellow Robin (*Eopsaltria australis*), Fuscous Honeyeater (*L. fuscus*), Grey Shrike-thrush (*Colluricincla harmonica*), Spotted Pardalote (*Pardalotus punctatus*), Superb Fairy-wren (*Malurus cyaeneus*), Weebill (*Smicronis brevirostris*), and Yellow-tufted Honeyeater (*L. melanops*).

**Table 1:** Classification of species according to their modelled response to tree-cover and their expected mobility.

Mobility	Response to landscape tree-cover	
	Decliner	Tolerant
Mobile	Fuscous Honeyeater (*Lichenostomus fuscus*; FH)	White-plumed Honeyeater (*Lichenostomus penicillatus*; WPH)
Moderate inconclusive^1^	Yellow-tufted Honeyeater (*Lichenostomus melanops*; YTH)	Striated Pardalote (*Pardalotus striatus*; STP)
	Spotted Pardalote (*Pardalotus punctatus*; SPP)	
	Grey Shrike-thrush (*Colluricincla harmonica*: GST)	
	Weebill (Smicornis brevirostris; WB)	
Sedentary inconclusive^1^	Eastern Yellow Robin (*Eopsaltria australis*; EYR)	
	Superb Fairy-wren (*Malurus cyaneus*; SFW)	
Sedentary	Brown Treecreeper(*Climacteris picumnus*; BT)	

1 For mobility, ‘inconclusive’ is used where there is uncertainty about mobility levels from the literature.

### Construction of landscape resistance models

The geographic area used for spatial modelling was the minimum convex polygon enclosing all of the sample points, with a 25 km buffer surrounding this polygon added to minimize the increase of resistance values due to the grid boundary [67]. We assigned a ‘no data’ value to cells outside of this area and excluded them from all calculations. All raster processing was carried out in ARCGIS version 9.3 [68] and the results output to ASCII grid format using the Export to Circuitscape Tool [69]. The scale of these raster data was chosen as the best compromise between the functional grain [29] considered most relevant to the birds (detectability of large individual trees and linear strips of tree-cover requiring 10 m resolution), data availability and the size of the grid (hence computational load).

### Landscape resistance surfaces were created as follows

#### (1) Null model surface

Two null models were applied. One assumed that there is no spatial structure to genetic differentiation due to unrestricted gene flow at the scale of the study area. There is no resistance surface for this model, as spatially random genetic variability is expected. A second null model assumed homogeneous resistance, i.e. the analogue of isolation-by-distance [50], for this model a raster with all cells having a resistance value of 1 was used. This surface allowed calculation of appropriate values that could then be used in partial Mantel tests to condition for the effect of geographic distance.

#### (2) Surfaces based on tree-cover

A 10 m resolution raster of vegetation cover >2 m height [64], essentially tree-cover for the study area, is of sufficient resolution to allow identification of large, isolated trees and contiguous tree-cover. The 10 m raster was generalized to 25 m (the finest scale at which all relevant datasets were available), such that any cell containing a 10 m tree pixel was identified as tree-cover. All cells of tree-cover were allocated a value of 1 and cells with no tree-cover were assigned a higher resistance value (2, 5, 10 or 100) to create four models of alternative resistance (Table 2). Models based on these surfaces were denoted TREE with a suffix for the resistance of the treed and non-treed area (e.g. TREE_1_5).

**Table 2:** Values used for resistance surfaces for developing each landscape model.

Model groups	Resistance surface/model code	Native tree-cover	Horticulture/pine	Unimproved pasture with scattered trees	Crop/improved pasture with scattered trees	Cleared land no scattered trees	Urban	All land-cover	Trees	Probable habitat	All other cells
Isolation-by-distance	UNIFORM							1			
Tree-cover	TREE_1_2								1		2
	TREE_1_5								1		5
	TREE_1_10								1		10
	TREE_1_100								1		100
Habitat suitability^1^	HAB_1_2									1	2
	HAB_1_10									1	10
Expert Opinion^2^	BT_EO_100	1	2000	1.2	1.2	2000	2000				
	BT_EO_5000	3.07	8000	4000	6000	8000	8000				
	EYR_EO_100	1	1.3	1.3	1.3	2000	2000				
	EYR_EO_5000	2000	6010	6010	8000	10000	10000				
	FH_EO_100	1	1.8	1	1	1	1.8				
	FH_EO_5000	2.17	2010	2010	2010	4010	4010				
	GST_EO_100	1	1.3	1.13	1.3	2000	1.8				
	GST_EO_5000	2.9	2000	7.17	2010	6010	6010				
	SFW_EO_100	1	1.02	2000	2000	2000	1.8				
	SFW_EO_5000	2000	6000	10000	10000	10000	10000				
	WB_EO_100	1	1.8	1.8	1.8	2000	1.3				
	WB_EO_5000	11.6	6010	4000	4000	8000	8000				
	WPH_EO_100	1	1	1	1	1	1				
	WPH_EO_5000	2.62	10.1	5.6	6.32	2010	7.45				
	YTH_EO_100	1	1.8	1	1	1	1.8				
	YTH_EO_5000	2.17	2010	2010	2010	6010	6010				

1 The habitat suitability models (HAB_1_2 and HAB_1_10) were run separately for each species (because the area and location identified as habitat is different for each species), but are included only once in this table as the same resistance values for habitat and other cells were used for all species.

2 Species codes for models are given in Table 1. The number at the end of the model code indicates the distance in metres over which resistance was estimated. Estimates for other distances, 200 m, 500 m, 1 km, 2 km and 10 km, which were not used in the final models, are available from the corresponding author on request.

#### (3) Surfaces based on habitat suitability derived from species distribution models (SDM)

The base data were represented by a 25 m raster of the predicted probability of occurrence of a species based on modelling presence records in relation to a range of spatially explicit environmental variables from satellite chrono-sequences, digital elevation models (for terrain and climate), and radiometric data [70]. The continuous SDM outputs were transformed to produce a binary result (i.e. part of or not part of the distribution of the species) employing a default threshold that maximises the diagnosticity measure [71]. Two models for each species with either high (10) or low (2) resistance for areas not classified as part of the species'distribution, were constructed and are referred to by the species abbreviation with a suffix of ‘HAB’.

#### (4) Surfaces based on bird species mobility in land cover classes, predicted by expert opinion

The dispersal behaviour of nearly all of the study species is poorly known, apart from the Brown Treecreeper [72] and Superb Fairy-wren [73]. We therefore sought expert opinion on it. Five ornithologists with expert field knowledge of the birds of the study area were asked to estimate, for each of the study species, the probability that an individual bird, during its lifetime, would traverse distances of 100 m, 200 m, 500 m, 1 km, 5 km or 10 km of a given land-cover class. The maximum value was 1 and the minimum permissible value was set at 0.0001. This was repeated for each of nine land-cover classes identified in a modelled GIS land-cover classification for the area (Sinclair SJ, White MD, Medley J, Smith E, Newell GR, Unpublished Manuscript). Two species, Spotted Pardalote and Striated Pardalote, were not included in the expert opinion elicitation because the decision to include them in the study post-dated the opinion survey.

In order to establish the among-expert variation in opinion, variance of estimates among experts and species as random effects were analysed. We used a linear mixed effects model and correlation of variance in the R package lmer4 [74], following [75]. Mean estimates of all experts for each combination of distance, land-cover class and species were calculated and used as a mean probability of dispersal (i.e., landscape conductivity). The reciprocal of this conductivity value, the land-cover class resistance, was to develop resistance surfaces and calculate effective distance for each species.

A 25 m raster of land-cover classes was derived from satellite imagery (Sinclair SJ, White MD, Medley J, Smith E, Newell GR, Unpublished Manuscript), with further categorization of cleared agricultural land with or without scattered trees. The final land cover classes were: (i) native tree-cover, (ii) plantation and horticulture, (iii) urban, (iv) unimproved pasture and native grassland, (v) improved pasture and (vi) arable crop. For the last three classes, all cells within 50 m radius of a tree pixel and not in contiguous tree-cover were denoted as scattered trees. These classes were assigned resistances according to the mean opinion of experts. Models based on these surfaces were denoted by the species abbreviation followed by EO (for Expert Opinion) and the distance for which conductivity was being estimated. For example, the model for Brown Treecreeper (BT) movement over 5000 m was denoted BT_EO_5000.

For all resistance surfaces, measures of effective distance between all 65 sampling points (Figure 1) were calculated with a) the least-cost path approach, using UNICOR Version 1.0 [76]; and b) isolation-by-resistance using Circuitscape version 3.5.1 employing the pairwise resistance and connection between eight cells options [44], [46], [47].

The existence of artificial boundaries in raster surfaces used for calculating isolation-by-resistance leads to inflation of resistance estimates [67]. Given that cells outside the model grid area were assigned an infinite resistance [47], there will be an increase in pairwise resistance between points close to the edge of the grid. We also considered the shape of the relationship between resistance, least-cost and linear distance on a bounded grid in comparison to the expectations of isolation by distance of either a linear or log-linear relationship with distance [77] again to inform null model choice.

### Correlations among models

Landscape models of effective distance resulted in pairwise matrices for the 65 sample sites. These data (2080 pairwise comparisons) were non-independent: each 65×65 matrix contained only 32 independent pairwise comparisons, the maximum possible without using a point twice. In order to compare alternative models while maintaining independence, correlation coefficients between landscape models were estimated by repeatedly sampling 32 randomly selected pairs for 1000 iterations of each of the pairwise distance matrices for each species and the tree-cover model. Mean estimated *R^2^* and the 95% intervals for each model in comparison with the null models (isolation-by-distance) were calculated. We used this approach rather than Mantel correlations because it provides an appropriate estimate of the true correlation among models conditional on the number of distinct data points (i.e. *N* = 32), rather than the much-inflated number associated with all pairwise correlations. Moreover, this bootstrapping technique provides an indication of potential variability in model correlations, which cannot be derived from the Mantel correlation. On the basis of this assessment, a subset of models including the appropriate null were chosen for ranking on prior expectation of their ability to predict genetic-distances between sample sites (to be tested in a later paper explicitly linked to this one).

### Forming the hypothesis: within-species ranking of the likelihood that landscape models will predict genetic data

Our models incorporate a range of heterogeneous landscape models implemented as isolation-by-resistance, and two null models: isolation-by-distance, where individuals' mobility and gene flow are restricted by geographic distance alone; and complete lack of significant spatial pattern at the scale of our study as individuals' mobility is unrestricted at the scale of the study area (i.e. panmixia).This last hypothesis is characterised by no significant effect of both isolation-by-resistance and isolation-by-distance.

Based on existing knowledge for each species derived from the major reference work on the avifauna of the region [65], [66] (Table S2), species' response to tree-cover change (tolerant or decliner) and expert opinion on species' mobility, we ranked the models on their ability to predict genetic structure. These rankings of models for each species represent our hypotheses. We based our ranking on the following.

1. The mobility of some species is sufficiently restricted to result in evidence of isolation-by-distance at the scale of the study, whereas more mobile species are not expected to show this effect (i.e. sufficient individuals move throughout the study area and cause gene flow to result in drift connectivity [18] and there will be no spatial pattern in their genetic variability).
2. We assume that habitat loss and fragmentation will reduce genetically effective dispersal between remnant tree-cover, especially for low-mobility ‘decliners’. If this proposition is correct, our model rankings are more likely to reflect the genetic data.

## Results

### Development of landscape resistance models

#### Null models and Circuitscape edge effect

**Figure 2:**
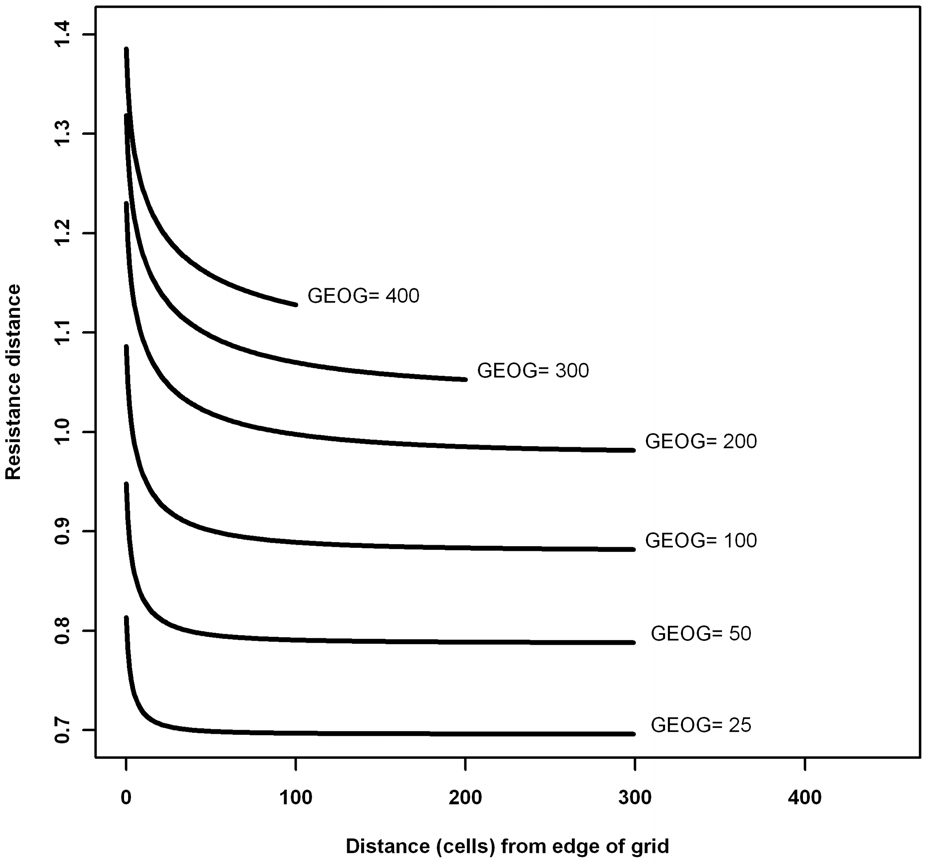
Pairwise resistance as a function of distance from the point nearest to the edge of the grid. Circuitscape isolation by resistance calculated over a linear distance in a circular grid of uniform resistance, 1 unit per cell, cell size 1 unit, and grid radius 500 cells. Each curve represents a different pairwise geographic distance. As a pairwise distance increases, so does the distance from the edge of the grid at which an edge effect of increased resistance distance is apparent. Where the edge of the grid represents an artificial barrier the resistance distance will be overestimated.

Values for pairwise least-cost distance and isolation-by-resistance (Circuitscape) across the study area when all grid cells had resistance equal to one (UNIFORM) were correlated with the geographic distance (GEOG) and with the log-transformed geographic distance (logGEOG). For least-cost distance on a uniform surface, the relationship with GEOG was strongest (R^2^ = 0.998). For Circuitscape on a uniform surface (UNIFORM), over all pairs, correlation was also strongest with GEOG, but for pairs separated by less than 50 km it was more highly correlated with logGEOG (all pairs: R^2^ = 0.97 and 0.89; pairs <50 km: 0.90 and 0.99 respectively). The cause of this complex curve is an ‘edge effect’ in Circuitscape, where pairwise resistances increase toward the edge of the grid. We demonstrated this ‘edge effect’ for a simplified simulated dataset (Figure 2) and for the more complex pattern of our study area (not shown). This effect is disproportionately larger for greater pairwise distances at the same geographic distance from the grid-edge (Figure 2). Consequently, for models based on least-cost distance, a suitable null model for comparison is simple geographic isolation (GEOG). For models developed using isolation-by-resistance (Circuitscape), the most appropriate null model is CS_UNIFORM i.e. the model developed using Circuitscape with a uniform surface that also incorporates the edge effect.

#### Expert opinion models

Over all distances combined, the variation in mobility estimates (i.e probabilities of traversing a given distance) among species was small (5% of variance in estimates) compared with variation among distances (28%), and was similar to variation among land use (9%) and experts (7%). When within-distance variation was considered, among-expert variance was the largest component of variance for distances ≤2 km (18–28%). At distances >2 km, land-use and species were attributed the greatest proportion of variance.

There was a bimodal distribution of mean estimates of resistance. Mean estimates were either ≤23 (low resistance), or >2000 (high resistance) in each species. Mean resistance estimated for the three agricultural land-covers without trees (i.e. unimproved pasture, improved pasture and arable land) were equal, as were crop and improved pasture with scattered trees. Therefore, the initial nine land-cover classes were reduced to six resistance classes (Table 2).

The ‘tolerant’ White-plumed Honeyeater differed from all other species. The estimate of land-cover resistance was low (<10) for up to 1 km for all land-covers, and for all distances for all land-covers except agricultural land without scattered trees. All other species submitted for expert opinion (all ‘decliners’) were estimated to have high resistance to movement (>2000) through land-cover classes other than tree-cover at distances ≥200 m.

The ranking of mean estimates by experts of movement ability through continuous tree-cover was similar to the classification of dispersal abilities based on the literature (Table S2). White-plumed Honeyeater, Yellow-tufted Honeyeater and Fuscous Honeyeater were estimated to have low resistance to movement up to 10 km, the maximum distance for which expert opinion was sought. For the other species, which we identified on the basis of the literature as poorer dispersers than the honeyeaters (Table S2), much higher resistance to movement through tree-cover over 2 km or greater distances was estimated by the experts. However, within these poorer dispersers there was disagreement on ranking. Literature suggests that Brown Treecreeper was the least mobile, followed by Eastern Yellow Robin Superb Fairy-wren and Grey Shrike-thrush. Expert opinion estimated Superb Fairy-wren and Eastern Yellow Robin as the least mobile (high resistance at ≥2 km in tree-cover), Brown Treecreeper, Grey Shrike-thrush, Weebill and were estimated to have high resistance only at ≥5 km in tree-cover.

We grouped the species according to information on their mobility summarised from the literature (Table S2) and expert opinion. For some species, the available information was inconclusive; for example, the species may be described as generally sedentary but with anecdotal evidence of longer distance movements or *vice versa*. We classified all species into four groups (Table 1): Sedentary/poor dispersers (Brown Treecreeper); species with inconclusive information that we considered were probably sedentary (Eastern Yellow Robin and Superb Fairy-wren); species with inconclusive information that we considered were probably of moderate or higher mobility (Spotted Pardalote, Grey Shrike-thrush, Striated Pardalote, Yellow-tufted Honeyeater and Weebill); and mobile species/better dispersers (Fuscous Honeyeater and White-plumed Honeyeater).

### Correlations among models

All but three models with heterogeneous landscape resistances were correlated with GEOG, logGEOG, and CS_UNIFORM (estimated *R^2^*>0.5, Table S3). These three models (EYR_HAB_10, EYR_EO5000 and SFW_EO5000) had the highest mean resistances (i.e. lowest predicted gene flows). Least-cost distance models had a higher estimated mean correlation with GEOG (mean *R^2^* = 0.95) than did isolation-by-resistance models with any of GEOG, logGEOG or CS_UNIFORM (mean *R^2^* = 0.73, 0.74 and 0.67 respectively, Table S3).

For the White-plumed Honeyeater, Yellow-tufted Honeyeater and Fuscous Honeyeater, the low resistance (EO_100 and EO_200) expert opinion models were indistinguishable from isolation-by-distance models (R^2^∼1, Table S3). Therefore, EO_100 and EO_200 were not used for predictions. For the high resistance model set, we chose EO_5000, as resistances for this distance showed the most discrimination among species and the highest proportion of variance in estimates (41% Table 3) due to the biologically pertinent factors of species and land-cover.

**Table 3:** Variance in expert opinion of land-cover resistance to the movement of bird species.

Variance component	All distances	Distance (m)						
		100	200	500	1000	2000	5000	10000
Distance	28							
Expert	7	18	18	22	28	19	8	14
Land-cover	9	4	4	5	9	18	21	25
Species	5	3	3	1	1	6	20	14
Residual	51	75	75	72	62	57	50	47

### Within-species ranking of models

Models were ranked, in the order of their predicted correlation with genetic distances, based on knowledge and expert opinion of the mobility and response to changed landcover for each species (Table 4, Table S2). Highest correlation was ranked first and lowest correlation seventh. The ranking resulted in six hypotheses for the 10 species. The most sedentary decliners (Brown Treecreeper, Eastern Yellow Robin, and Superb Fairy-wren) were ranked similarly with high resistance models expected to provide best fit. Two moderately mobile decliners (Spotted Pardalote and Grey Shrike-thrush) were also ranked similarly. Weebill and Yellow-tufted Honeyeater were ranked similarly. Fuscous Honeyeater, the most mobile species, but a decliner, had an idiosyncratic response to landscape configuration: no isolation-by-distance was predicted, but it may still show weak structure due to loss of connectivity in spite of its apparent mobility. The two tolerant species (Striated Pardalote and White-plumed Honeyeater) were not expected to have responses correlated with landscape heterogeneity. On balance, the information for White-plumed Honeyeater suggested that it may not be as highly mobile as the other honeyeaters and thus may show weak isolation-by-distance. The information on mobility levels for Striated Pardalote was inconclusive, and therefore we ranked isolation-by-distance and panmixia equally.

**Table 4:** Predicted rank^1^ of correlation coefficients between landscape models and genetic distances.

Model	Species attributes/ requirements for better fit to model	Species^2^									
		BT	EYR	FH	GST	SFW	SPP	STP	WB	WPH	YTH
TREE_1_2	Weak isolation-by-resistance	4 =	4 =	1 =	1 =	4 =	1 =	3 =	1 =	3 =	1 =
TREE_1_10	Isolation-by-resistance and strong isolation-by-distance	2 =	2 =	4 =	4 =	2 =	4 =	3 =	3 =	3 =	3 =
HAB_1_2	Isolation-by-resistance HAB model provides better identification of suitable dispersal habitat than trees alone	4 =	4 =	1 =	1 =	4 =	1 =	NA	1 =	3 =	1 =
HAB_1_10		2 =	2 =	4 =	4 =	2 =	4 =	NA	3 =	3 =	3 =
EO_5000	Scattered trees important coupled with strong isolation-by-distance	1	1	6	4 =	1	NA	NA	3 =	3 =	3 =
No spatial structuring/ panmixia	Highly mobile, ‘Tolerant’	7	7	3	7	7	6	1 =	6	2	6
Isolation-by-distance only rank^3^		6	6	7	3 =	6	3	1 =	7	1	7
Isolation by distance strength^3^		Strong	Strong	None	Weak	Strong	Weak	Weak	Weak	Weak	None

1 Species codes are given in Table 1.

2 For each species, models are ranked from highest (1) to lowest (7).

3 The row ranking isolation-by-distance has a rank for the occurrence of isolation-by-distance alone, and ‘strong, weak or none’ for the strength of the isolation-by-distance signal expected, whether or not isolation-by-resistance is also present.

## Discussion

We made predictions about the likely genetic response of 10 bird species to the landscapes used in the study based on available data and on expert opinion. We grouped the ten species into seven groups for expected response. Hypotheses were framed as the ranking of a series of landscape distance matrices (uniform resistance (isolation-by-distance), and heterogeneous isolation-by-resistance/least-cost distance) plus no spatial structure for panmixia, for testing against genetic distances.

We contend that the *a priori* ranking of a set of alternative landscape distance models based on available ecological information is a robust approach to testing landscape genetic hypotheses. This may be even more important in the light of problematically correlated landscape models and the risk of spurious correlations [49], [51]. Ranking of multiple species adds generality. Prior predictions explicitly link characteristics of the organisms to their response to landscape structure [23] and are considered to offer a more rigorous test of inferences about ecological processes [22], [78].

### Application of expert opinion and descriptive literature

The low variance in expert opinion among species suggests that experts believed that the loss of structural connectivity has a similar effect on the mobility of nearly all species. However, the White-plumed Honeyeater stood out as the exception as might be expected for the one tolerant species for which we had expert opinion. Some experimental evidence exists for the Brown Treecreeper and to a lesser extent for Eastern Yellow Robin, White-plumed Honeyeater, Fuscous Honeyeater and Grey Shrike-thrush, that movement is constrained by cleared gaps of 100–200 m in tree-cover, but may be facilitated by scattered trees in the intervening space [72], [79]. This pattern was reflected in the expert opinion of relative mobility through land-covers with and without scattered trees over a distance of 100 m for all species except the three honeyeaters.

Our assessments of the mobility of the different species were based on sparse datasets, mostly inferred from descriptive material and expert opinion. This enabled us to develop simple hypotheses that distinguish the expected landscape responses of a group of passerines found in the same general vegetation type but showing markedly different response to habitat loss.

Expert opinion was consistent with descriptive information from standard reference sources [65], [66] in the grouping of birds' mobility. However, it did not provide strong discrimination among most of the species in terms of response to structural connectivity. Gap-crossing behaviour may be similar for species that we have identified as having widely varying mobility [72]. If so, then our predictions of responses to heterogeneous tree-cover would not be supported, and response to tree-cover gaps should be similar in all woodland-dependent species. Our predictions of isolation-by-distance, which are determined by general mobility rather than gap-crossing behaviour, would be unaffected.

Garrard et al. [80] developed a model of natal dispersal based on feeding guild, wing length, mass and existing natal dispersal data reviewed from five studies of 84 (mainly northern hemisphere) species in 12 avian orders. The model was then used to predict median natal dispersal distance for the species studied by Radford et al. [60]. A negative relationship was found between natal dispersal distance and the effects of habitat fragmentation on prevalence of a species in the landscape. This agreed with our predictions - that the effects of habitat fragmentation will be greater for poorer dispersers. However, the individual species identified as having the shortest natal dispersal distances by Garrard et al. [80] are those identified here as the most mobile (the honeyeaters *Lichenostomus* spp.). We believe this disagreement arises from the feeding guild classification of ‘omnivore’ being inappropriate for nectarivorous/insectivorous honeyeaters that are more prominent in the south-eastern Australia avifauna [81] than in the Garrard et al. [80] dataset.

### Comparison among species and choice of null models

The consideration of multiple species allowed the ranked expectations per species to be contrasted. This offers additional inferences to the absolute fit of predictions to the sampling design, and has been highlighted as a way to enhance the usefulness of landscape-genetic studies [23].

The extent and scale of the grid for Circuitscape calculations is limited practically by computational capacity (memory and time) and data availability for land-cover [67], and leads to grid ‘edge effect’ (Figure 2). The edge effect in Circuitscape computation enables isolation-by-resistance to account for complex range or habitat shapes in modelling of genetic differentiation [44]. However, where the Circuitscape grid has artificial boundaries that are imposed due to data or computational limits, this edge effect must be accounted for, and minimized through buffering [67]. Therefore, we recommend the CS_UNIFORM distance as the null model (effect of isolation-by-distance) for comparisons with other Circuitscape resistances when considering heterogeneous landscape connectivity, and particularly for use in partial Mantel tests. CS_UNIFORM distance most closely follows the predictions of isolation-by-distance at multiple distances and in different habitat configurations [44], [82], and alleviates the inflation of resistances caused by artificial boundaries [67].

The shared basis of all the models of land-cover classes, and principally tree-cover, along with the relatively low resistance differentials, means that nearly all the models are correlated (Table S3), making them difficult to distinguish among. The high level of correlations between plausible resistance models is near universal. Causal modelling provides a robust methodology for comparison of, and selection among correlated distance hypotheses [49], particularly when coupled with cross-conditioning of competing models [56]. McRae [44] argued that the value of the isolation-by-resistance model lies in its ability to examine the more subtle effects of dissimilar gene flow through different landscape components. Lower mean correlations between isolation-by-resistance compared to least-cost distance for the same resistance surfaces provides a greater level of discrimination in pairwise comparisons across complex landscapes than do least-cost distances. Therefore, for a given set of resistance estimates, an isolation-by-resistance model may be more readily distinguished from other models, and from isolation-by-distance models.

### Resistance values in this system compared with others

The resistance values identified here, with the exception of some of the expert opinion models (Table 2), are at the lower end of those published employing least-cost path [30], [36], [37], [39], [41] or isolation-by-resistance [55]. Some of these authors used values as low as 1∶2 for their habitat∶ matrix ratio; 1∶10 to 1∶1000 were more usual, while 1∶10,000 to 1∶100,000 were used as barriers. The studies cited above all involved fragmentation impacts on mammals and amphibians. Birds may be expected to experience lower levels of landscape resistance because flight allows them to cross gaps more rapidly and to cover larger distances more efficiently than non-volant terrestrial vertebrates. The one recent study that used cost distance to examine landscape effects on passerine genetic structure used resistance ratios similarly low to ours [27]. The extremes in those models varied from 1∶2 to 1∶4 in a least-path distance model.

Other multi-model selection studies have sought to maximise the explanatory power of the best model through a multi-step approach, first optimising the contribution of individual landscape elements, and then combining them [55], or have combined inferences from extensive tracking data and to determine the most plausible landscape surfaces, that were then combined to produce a large number of combination models for a single species [54], [56], [83]. By making prior predictions between species comparisons using the qualitative data available on each, we have taken a different approach compared to previous studies to maximise the strength of our inferences. This approach is most useful where multiple species are sampled concurrently (e.g such as mist-netting of passerines), and where there are not extensive data on individual movements, though basic descriptive natural history is available. The study system did not have the mountainous terrain, extreme climate and differentiation of forest types present in the previous studies of mammals in the mountains of the north –western USA [53]–[56], [83]. The most similar approach to date [28] was also on forest birds, although that study was comparing the expected response of a habitat generalist with a specialist in largely continuous forest.

### Maximising the ability to discriminate between correlated models

It might be expected that isolation-by-resistance will accumulate over distance, resulting in stronger signals over greater distances. However, in a fragmented landscape these greater distances also increase the number and importance of alternative routes and the number of and complexity of configuration landscape elements that individuals (or gene flow) encounter. One recent set of simulations has suggested that this additional complexity with distance may obscure effects, and, perhaps counter-intuitively, landscape resistance signals may be more prominent at short distances [84]. However other recent simulations across landscapes of equal size, but varying in complexity and cover, found that the best fit of genetic data and landscape resistance was in landscapes with low but aggregated cover and intermediate connectivity (Graves et. al, Unpublished Manuscript). The comparison of, and discrimination among, correlated models may result in increased Type-1 error rates [51]. Use of ‘two stage causal modelling’ [49], [56], along with separate testing between landscapes of differing cover and aggregation levels at short distance (within landscapes) and longer distance (across study area), in our subsequent testing of predictions with genetic data may help clarify some of these issues. Ultimately, to distinguish unequivocally among correlated landscape models may require extensive, spatially-explicit population-genetic and demographic simulations across a range of landscape arrangements and relative resistance values, and the development of more powerful statistical techniques to deal with the necessarily pairwise data of landscape genetics [19], [45], [85].

We have documented a process for making explicit predictions of expected genetic outcomes for a range of species in a system of conservation concern within and among landscapes based on available data. The process maximises the inferences that can be made about landscape connectivity effects for the system. Our model study system, widespread and relatively abundant birds, means that we have been able to gather good sample sizes for genetic analyses across multiple species. However, this is countered by their high mobility compared with many other organisms, and the small proportion of the populations that we have been able to sample – a result of sampling of many landscape units. Use of prior prediction ensures that the study tests, and if possible extends, our knowledge of the biological reality of connectivity in the system. If we can detect effects in this system, then the presented approach is very likely to be more effective for less-mobile species with smaller population sizes. Ideally we would be able to identify a best model for each species. However if we are able to identify a group of related models, this is likely to determine the importance of connectivity effects for the less well-connected species. This may be sufficient to develop management recommendations for the system as a whole.

## Supporting Information

Material S1 Classification of species**'** occurrence in response to landscape tree-cover.
(PDF)

Table S1 Table S1 AICc values for each model and change point threshold in tree-cover.
(PDF)

Table S2 Mobility information from HANZAB [65], [66].
(PDF)

Table S3 Mean correlation between, and credible intervals for, isolation models.
(PDF)
