# Supplementary material for: Predicting Landscape-Genetic Consequences of Habitat Loss, Fragmentation and Mobility for Multiple Species of Woodland Birds
Source: PLoS One. 2012 Feb 17;7(2):e30888. doi: 10.1371/journal.pone.0030888 (PMC3281894; doi:10.1371/journal.pone.0030888)
Supplement: Material S1 — Classification of species' occurrence in response to landscape tree-cover. (PDF) [file pone.0030888.s001.pdf]

S1. Classification of species' occurrence in response to landscape tree-cover

Methods

We used data from Radford et al. [1] to identify 'decliner' and 'tolerant' bird species. Species in which landscape-level incidence decreased disproportionately to landscape-level tree-cover were classified as 'Decliner' species and were identified by a positive relationship between incidence and tree cover. Species in which landscape-level incidence was proportionate to, or increased disproportionately to, landscape-level tree cover were classified as 'Tolerant' species and were identified by a null or negative relationship between incidence and tree cover.

There were 10 survey sites in each landscape, distributed within remnant tree-cover. Each site was surveyed four times over the course of 12 months. Species incidence per landscape was therefore a score out of 40. The incidence of 58 species in each of the 24 landscapes[1] was modelled as a function of landscape-level tree-cover (TREE). For each species, seven models were fitted using least squares regression in Genstat V.10 [2]. We fitted the null model (intercept only, zero slope), four 'continuous' models (linear, logarithmic, quadratic and power) and two 'threshold' models (piecewise and change-point). Threshold models separate the response variable into two relatively homogeneous groups either side of a threshold value in the environmental gradient (tree-cover in this case). In piecewise regression, the slope of a regression fitted independently to the groups either side of the threshold may vary, whereas in change-point analysis the slope of the groups either side of the threshold is always zero (i.e. the groups are characterised by a different mean and deviance).

When the response data are divided into two groups, the sum of the deviance for the two sub-group is always less than or equal to the deviance of the entire data [3]. Therefore, each possible threshold (i.e. value in the range of the environmental variable) is associated with a deviance reduction. To identify the the threshold in landscape-level tree cover that maximises the deviance reduction for the piecewise (T_{pw}) and change-point (T_{cp}) models, sequential values of tree-cover (from 0 to 60% tree cover) were fitted and the value with lowest residual deviance identified as the threshold.

To reduce heteroscedasticity, species incidence was first weighted by 1/(variance + 0.5), where variance was calculated for sequential groups of four landscapes (ordered by increasing tree-cover). The best model for each species among the seven candidate models was selected using AIC_c. The models fitted were:

1. Null (intercept only): y = mean(y)
2. Linear: $y = \beta_0 + \beta_1 * TREE$
3. Logarithmic: $y = \beta_0 + \beta_1 * Log_{10}(TREE)$
4. Quadratic: $y = \beta_0 + \beta_1 * TREE + \beta_2 * TREE^2$
5. Power: $y = \beta_0 + \beta_1 * TREE^{\beta 2}$
6. Piecewise: $y = \beta_0 + \beta_1 * TREE$ where $TREE < T_{pw}$; $y = \beta_0 + \beta_1 * TREE + \beta_2 * (TREE - T_{pw})$ where $TREE > T_{pw}$ [4]
7. Binomial change-point: $y = \beta_0 + \beta_1 * T_{term}$; where $TREE < T_{cp}$, $T_{term} = 0$; where $TREE > T_{cp}$, $T_{term} = 1$ [5]

Surveys were conducted only within remnant tree-cover in each landscape [6]. This means that the null model represents a proportionate decline in number of birds with landscape tree-cover; that is, incidence in a patch of suitable habitat is not related to the overall proportion of tree cover in that landscape. Thus, species in which the null model was selected as the best fit were identified as 'tolerant'. Any species in which incidence increased disproportionately with decreasing landscape-level tree cover (i.e., any one of the other six models was selected with a negative coefficient) was also identified as 'tolerant'. Any species in which incidence decreased disproportionately with decreasing landscape tree cover (i.e., any one of the other six models was selected with a positive co-efficient) was identified as a 'decliner'.

Results

Of the 58 species, the null model fitted best for 21 species, the linear model for 6 species, the logarithmic model for 4 species, the quadratic model for 4 species and the change-point model for 23 species. For two species (Tree Martin *Hirundo nigricans* and Striated Pardalote *Pardalotus striatus*), the change-point model was selected but with a negative co-efficient indicating higher incidence in landscapes with lower tree cover, so these two species were considered 'tolerant'.

Following consideration of abundance and evenness of distribution across the study landscapes (see main text) ten study species were chosen: two 'tolerant' species and eight 'decliners'. The two tolerant species were the White-plumed Honeyeater (*Lichenostomus penicillatus*), for which the null model was selected, and the Striated Pardalote, best described by the change-point model with a negative coefficient (Table S1). Of the eight 'decliners', the change-point model was selected for six species – Fuscous Honeyeater (*L. fuscus*), Grey Shrike-thrush (*Colluricincla harmonica*), Spotted Pardalote (*Pardalotus punctatus*), Superb Fairy-wren (*Malurus cyaeneus*), Weebill (*Smicronis brevirostris*) and Yellow-tufted Honeyeater (*L. melanops*). The Eastern Yellow Robin (*Eopsaltria australis*) showed a linear relationship. The quadratic model provided the best fit for the Brown Treecreeper (*Climacteris picumnus*) with highest incidence recorded in mid-cover (~30%) landscapes (Supplementary Material Table S2). The decline in incidence of the Brown Treecreeper in landscapes above 30% tree cover was probably due to absence of suitable habitat (particularly hollow-bearing trees).

Table S1 AICc values and change point threshold in tree-cover for species incidence model fitting.

Species	Model							
	Null	Linear	Log	Quad	Power	BS	CP	Change Point threshold value (%)
Brown Treecreeper	10.5	12.2	8.1	4.9	9.3	5.4	7.9	NA
Eastern Yellow Robin	26.0	4.1	13.6	5.5	6.6	7.3	5.1	NA
Fuscous Honeyeater	25.5	7.2	11.9	7.4	9.0	9.4	6.4	17.0
Grey Shrike-thrush	24.8	25.3	17.4	17.9	15.7	14.9	12.9	5.2
Superb Fairy-wren	7.5	2.8	2.4	4.5	4.9	7.1	1.9	18.1
Spotted Pardalote	22.8	3.7	4.2	1.2	3.6	4.1	-2.0	11.7
Striated Pardalote	4.0	4.7	3.8	6.7	6.7	8.0	3.3	9.9
Weebill	13.2	5.8	3.0	7.0	5.9	7.8	2.7	8.4
White-plumed Honeyeater	5.9	8.4	8.0	7.5	9.9	7.0	6.1	NA
Yellow-tufted Honeyeater	27.5	8.1	22.6	11.0	11.1	10.6	-4.4	7.8

References

1. Radford JQ, Bennett AF (2007) The relative importance of landscape properties for woodland birds in agricultural environments. Journal of Applied Ecology 44: 737-747.
2. Payne RW, Murray DA, Harding SA, Baird DB, Soutar DM (2007) GenStat for windows. 10th Edn. ed: VSN International Ltd., Hemel Hempstead, UK.
3. Qian SS, King RS, Richardson CJ, (2003) Two statistical methods for the detection of environmental thresholds. Ecological Modelling : 166: 87-97.
4. Toms JD, Lesperance ML (2003) Piecewise regression: A tool for idnentifying ecological thresholds. Ecology 84: 2034-2041.

5. Siegel S (1988) Nonparametric statistics for the behavioral sciences; Castellan NJ, editor. New York: McGraw-Hill.
6. Radford JQ, Bennett AF, Cheers GJ (2005) Landscape-level thresholds of habitat cover for woodland-dependent birds. Biological Conservation 124: 317-337.
