# Supplementary material for: Predicting Landscape-Genetic Consequences of Habitat Loss, Fragmentation and Mobility for Multiple Species of Woodland Birds
Source: PLoS One. 2012 Feb 17;7(2):e30888. doi: 10.1371/journal.pone.0030888 (PMC3281894; doi:10.1371/journal.pone.0030888)
Supplement: Table S1 — AICc values for each model and change point threshold in tree-cover. (PDF) [file pone.0030888.s002.pdf]

Table S1 AICc values and change point threshold in tree-cover for species' incidence model fitting.

Species	Model							
	Null	Linear	Log	Quadratic	Power	Broken stick	Change point	Change Point threshold value (%)
Brown Treecreeper	10.5	12.2	8.1	4.9	9.3	5.4	7.9	NA
Eastern Yellow Robin	26.0	4.1	13.6	5.5	6.6	7.3	5.1	NA
Fuscous Honeyeater	25.5	7.2	11.9	7.4	9.0	9.4	6.4	17.0
Grey Shrike-thrush	24.8	25.3	17.4	17.9	15.7	14.9	12.9	5.2
Superb Fairy-wren	7.5	2.8	2.4	4.5	4.9	7.1	1.9	18.1
Spotted Pardalote	22.8	3.7	4.2	1.2	3.6	4.1	-2.0	11.7
Striated Pardalote	4.0	4.7	3.8	6.7	6.7	8.0	3.3	9.9
Weebill	13.2	5.8	3.0	7.0	5.9	7.8	2.7	8.4
White-plumed Honeyeater	5.9	8.4	8.0	7.5	9.9	7.0	6.1	NA
Yellow-tufted Honeyeater	27.5	8.1	22.6	11.0	11.1	10.6	-4.4	7.8
