# Supplementary material for: Predicting Landscape-Genetic Consequences of Habitat Loss, Fragmentation and Mobility for Multiple Species of Woodland Birds
Source: PLoS One. 2012 Feb 17;7(2):e30888. doi: 10.1371/journal.pone.0030888 (PMC3281894; doi:10.1371/journal.pone.0030888)
Supplement: Table S2 — Mobility information from HANZAB [65], [66]. (PDF) [file pone.0030888.s003.pdf]

Table S2, Mobility information from HANZAB [1,2]

Species	HANZAB précis	Mobility description and interpretive comments	Mobility classification
Brown Treecreeper	Sedentary. Some local dispersal, usually to adjacent territories. From 1953-1984, 1482 birds banded: 7 recoveries away from original site of capture, all < 10 km	Sedentary, sex-biased dispersal: males rarely disperse more than a few territories from natal site. Sedentary behaviour is corroborated by combination of high recovery rate, and no recoveries at > 8 km. Additional data radio-tracking and recapture data (V Doerr, unpublished) corroborate this view.	Sedentary
Eastern Yellow Robin	Mainly sedentary or resident - some seasonal movements, particularly from higher altitude to plains in south-eastern Australia in winter.	Mainly sedentary: inconclusive data. We believe EYR resident and sedentary within the study area. However, evidence of seasonal movements (often involving move to lower altitudes), and apparent influx of birds to some sites in the study area during winter. These may obscure underlying genetic structure of any resident population units, or, if some of these birds settle and interbreed, that may result in little or no isolation-by-distance at the study-wide scale.	Sedentary/ Inconclusive
Fuscous Honeyeater	Resident/ partly resident, local abundance varies with influx when nectar plentiful, movements in and out of areas sometimes noted. Perhaps partially nomadic.	Mobile, irregular seasonal movements with circumstantial evidence of large-scale movements (100s of km) in response to nectar resources. Only very small numbers present in some months during the field component of this project (Unpublished data)	Mobile
Grey Shrike-thrush	Sedentary/Resident with some local post-breeding dispersal. Described as nomadic or increasing in abundance in winter in some areas. Altitudinal movements from Alps in winter.	Inconclusive, mostly sedentary, however GST is a strong flyer, and combination of seasonal movements with post-breeding dispersal over unknown distances may weaken any isolation-by-distance.	Moderate/ inconclusive
Superb Fairy Wren	Sedentary. dispersal of young, and, rarely, of female breeders over short distances.		Sedentary

Spotted Pardalote	Apparently resident or sedentary in most of range though some regular possibly migratory movement is in SE Aus. Variously considered mainly sedentary, resident or partly migratory. Movement inland or North from higher elevations in SE of range in winter..	Inconclusive, recorded as resident in habitat similar to the study area (Chiltern, to the East of our study, and also at Creswick, well to the West).	Moderate/ inconclusive
Striated Pardalote	Resident, migratory or dispersive, *P .s. ornatus* and *P. s. substriatus* in temperate areas resident dispersive or migratory, some inland Northward movement in winter in south. of range .	.	Mobile
Weebill	Considered resident throughout range, some local movement, no regular seasonal movements.		Moderate/ inconclusive
White-plumed Honeyeater	Resident or sedentary, juveniles sometimes disperse widely from natal area, some (mainly local) movements to water in dry periods. No large-scale seasonal movements.	Moderately mobile, probably less so than Fuscous Honeyeater.	Mobile
Yellow-tufted Honeyeater	Mainly resident or sedentary, with local movements, possibly larger-scale movements (possibly in response to drought). *L.c. meltoni* most mobile, post-breeding dispersal in autumn or winter.	Inconclusive, possibly moderately mobile. *L.c. meltoni*. the subspecies resident in the study area is anecdotally identified as most mobile subspecies.	Moderate/ inconclusive

1. Higgins PJ, Peter JM, Steele WK, editors (2001) Handbook of Australian, New Zealand and Antarctic Birds. Volume 5: Tyrant-flycatchers to Chats. Melbourne: Oxford University Press
2. Higgins PJ, Peter JM, editors (2002) Handbook of Australian, New Zealand and Antarctic Birds. Volume 6: Pardalotes to Shrike-thrushes. Melbourne: Oxford University Press
