# Supplementary material for: Predicting Landscape-Genetic Consequences of Habitat Loss, Fragmentation and Mobility for Multiple Species of Woodland Birds
Source: PLoS One. 2012 Feb 17;7(2):e30888. doi: 10.1371/journal.pone.0030888 (PMC3281894; doi:10.1371/journal.pone.0030888)
Supplement: Table S3 — Mean correlation between, and credible intervals for, isolation models. (PDF) [file pone.0030888.s004.pdf]

**Table S3 Mean correlation between and credible intervals for isolation models.**

| | Isolation-by-resistance\ Circuitscape vs | | | Least-cost distance vs | |
|---|---|---|---|---|---|
| Model/Resistance Surface | UNIFORM | GEOG | logGEOG | GEOG | Isolation- by resistance |
| Correlation of Uniform/Isolation by distance Models | | | | | |
| UNIFORM | | 0.97 (0.902, 0.993) | 0.903 (0.868, 0.937) | 0.998 (0.997, 0.999) | |
| GEOG | | | 0.809 (0.653, 0.927) | | |
| logGEOG | | | | 0.807 (0.638, 0.926) | |
| Generic Treecover Models | | | | | |
| TREE_25_10 | 0.477 (0.088, 0.791) | 0.474 (0.095, 0.784) | 0.425 (0.051, 0.734) | 0.927 (0.889, 0.958) | 0.571 |
| TREE_25_100 | 0.327 (0.012, 0.697) | 0.331 (0.012, 0.702) | 0.294 (0.006, 0.632) | 0.881 (0.822, 0.929) | 0.419 |
| TREE_25_2 | 0.818 (0.595, 0.937) | 0.801 (0.586, 0.926) | 0.737 (0.487, 0.891) | 0.989 (0.982, 0.994) | 0.862 |
| TREE_25_5 | 0.577 (0.188, 0.837) | 0.57 (0.193, 0.833) | 0.515 (0.135, 0.785) | 0.949 (0.922, 0.971) | 0.666 |
| Sedentary decliners | | | | | |
| BTC_EO_100 | 0.991 (0.976, 0.997) | 0.966 (0.908, 0.992) | 0.905 (0.845, 0.958) | 0.999 (0.998, 0.999) | 0.964 |
| BTC_EO_5000 | 0.636 (0.229, 0.926) | 0.638 (0.25, 0.92) | 0.575 (0.157, 0.844) | 0.918 (0.876, 0.955) | 0.734 |
| BTC_HAB_10 | 0.827 (0.577, 0.959) | 0.831 (0.608, 0.957) | 0.751 (0.491, 0.907) | 0.921 (0.875, 0.96) | 0.825 |
| BTC_HAB_2 | 0.961 (0.902, 0.991) | 0.943 (0.871, 0.984) | 0.874 (0.784, 0.945) | 0.994 (0.99, 0.997) | 0.944 |
| EYR_EO_100 | 0.979 (0.945, 0.994) | 0.955 (0.892, 0.987) | 0.893 (0.823, 0.952) | 0.998 (0.997, 0.999) | 0.956 |
| EYR_EO_5000 | 0.236 (0, 0.788) | 0.241 (0, 0.815) | 0.214 (0.001, 0.704) | 0.817 (0.724, 0.889) | 0.442 |
| EYR_HAB_10 | 0.469 (0.078, 0.787) | 0.461 (0.077, 0.783) | 0.416 (0.05, 0.729) | 0.908 (0.857, 0.948) | 0.541 |
| EYR_HAB_2 | 0.816 (0.61, 0.939) | 0.796 (0.58, 0.925) | 0.735 (0.51, 0.889) | 0.985 (0.977, 0.992) | 0.850 |
| SFW_EO_100 | 0.987 (0.966, 0.996) | 0.962 (0.904, 0.99) | 0.901 (0.837, 0.955) | 0.998 (0.998, 0.999) | 0.961 |
| SFW_EO_5000 | 0.254 (0, 0.889) | 0.258 (0, 0.909) | 0.232 (0, 0.826) | 0.845 (0.779, 0.9) | 0.120 |
| SFW_HAB_10 | 0.587 (0.214, 0.841) | 0.591 (0.221, 0.845) | 0.539 (0.142, 0.791) | 0.905 (0.853, 0.947) | 0.652 |
| SFW_HAB_2 | 0.891 (0.746, 0.963) | 0.877 (0.724, 0.961) | 0.814 (0.649, 0.919) | 0.99 (0.984, 0.995) | 0.896 |
| Intermediate/Equivocal Decliner | | | | | |
| GST_EO_100 | 0.991 (0.976, 0.998) | 0.966 (0.908, 0.992) | 0.905 (0.845, 0.958) | 0.999 (0.998, 0.999) | 0.964 |
| GST_EO_5000 | 0.624 (0.204, 0.923) | 0.627 (0.227, 0.919) | 0.565 (0.135, 0.842) | 0.913 (0.868, 0.952) | 0.736 |
| GST_HAB_10 | 0.596 (0.198, 0.86) | 0.597 (0.205, 0.869) | 0.545 (0.155, 0.808) | 0.895 (0.838, 0.941) | 0.681 |
| GST_HAB_2 | 0.891 (0.722, 0.969) | 0.874 (0.694, 0.961) | 0.814 (0.633, 0.919) | 0.989 (0.982, 0.994) | 0.905 |
| SPP_HAB_10 | 0.516 (0.112, 0.842) | 0.519 (0.123, 0.853) | 0.468 (0.078, 0.765) | 0.906 (0.85, 0.947) | 0.611 |
| SPP_HAB_2 | 0.861 (0.688, 0.965) | 0.844 (0.672, 0.959) | 0.783 (0.58, 0.907) | 0.986 (0.978, 0.992) | 0.884 |
| WB_EO_100 | 0.989 (0.973, 0.997) | 0.905 (0.846, 0.958) | 0.964 (0.893, 0.99) | 0.999 (0.998, 0.999) | 0.963 |
| WB_EO_5000 | 0.595 (0.163, 0.927) | 0.598 (0.166, 0.927) | 0.542 (0.1, 0.857) | 0.903 (0.855, 0.946) | 0.727 |
| WB_HAB_10 | 0.599 (0.195, 0.899) | 0.606 (0.193, 0.913) | 0.544 (0.145, 0.837) | 0.892 (0.832, 0.94) | 0.715 |
| WB_HAB_2 | 0.899 (0.729, 0.979) | 0.886 (0.714, 0.966) | 0.816 (0.645, 0.923) | 0.99 (0.984, 0.995) | 0.917 |
| Mobile Decliners | | | | | |
| FH_EO_100 | 1 (1, 1) | 0.974 (0.913, 0.996) | 0.915 (0.865, 0.963) | 0.998 (0.997, 0.999) | 0.969 |
| FH_EO_5000 | 0.688 (0.304, 0.935) | 0.685 (0.322, 0.926) | 0.62 (0.234, 0.855) | 0.944 (0.914, 0.97) | 0.762 |
| FH_HAB_10 | 0.652 (0.281, 0.932) | 0.651 (0.28, 0.929) | 0.583 (0.2, 0.857) | 0.907 (0.855, 0.951) | 0.681 |
| FH_HAB_2 | 0.918 (0.798, 0.983) | 0.899 (0.77, 0.974) | 0.826 (0.674, 0.922) | 0.986 (0.978, 0.992) | 0.908 |
| YTH_EO_100 | 1 (1, 1) | 0.974 (0.913, 0.996) | 0.915 (0.865, 0.963) | 0.998 (0.997, 0.999) | 0.969 |
| YTH_EO_5000 | 0.641 (0.24, 0.937) | 0.641 (0.237, 0.936) | 0.581 (0.165, 0.861) | 0.924 (0.883, 0.958) | 0.750 |
| YTH_HAB_10 | 0.631 (0.231, 0.948) | 0.63 (0.233, 0.941) | 0.567 (0.145, 0.878) | 0.904 (0.849, 0.948) | 0.696 |
| YTH_HAB_2 | 0.914 (0.786, 0.985) | 0.895 (0.764, 0.977) | 0.826 (0.661, 0.925) | 0.989 (0.983, 0.994) | 0.913 |
| Mobile Tolerant | | | | | |
| WPH_EO_100 | 1 (1, 1) | 0.974 (0.912, 0.996) | 0.915 (0.865, 0.963) | 0.998 (0.997, 0.999) | 0.969 |
| WPH_EO_5000 | 0.784 (0.504, 0.957) | 0.772 (0.492, 0.952) | 0.709 (0.413, 0.891) | 0.974 (0.96, 0.985) | 0.818 |
| WPH_HAB_10 | 0.795 (0.551, 0.936) | 0.801 (0.567, 0.944) | 0.732 (0.47, 0.888) | 0.944 (0.912, 0.973) | 0.828 |
| WPH_HAB_2 | 0.957 (0.894, 0.988) | 0.942 (0.867, 0.984) | 0.876 (0.79, 0.951) | 0.996 (0.993, 0.998) | 0.947 |
| Mean | 0.75 | 0.736 | 0.680 | 0.949 | 0.782 |

Lower and Upper 95% credible intervals are given in brackets after mean pearson $R^2$
